# Applications of Sustainable Polymer-Based Phase Change Materials in Mortars Composed by Different Binders

**DOI:** 10.3390/ma12213502

**Published:** 2019-10-25

**Authors:** Mariaenrica Frigione, Mariateresa Lettieri, Antonella Sarcinella, José Luìs Barroso de Aguiar

**Affiliations:** 1 Innovation Engineering Department, University of Salento, Prov.le Lecce-Monteroni, 73100 Lecce, Italy; antonella.sarcinella@unisalento.it; 2 Institute of Archaeological Heritage-Monuments and Sites, CNR-IBAM, Prov.le Lecce-Monteroni, 73100 Lecce, Italy; mariateresa.lettieri@cnr.it; 3 Civil Engineering Department, University of Minho, Campus de Azurém, 4800-058 Guimarães, Portugal; aguiar@civil.uminho.pt

**Keywords:** cement, gypsum, hydraulic lime, mechanical properties, mortars, phase-change materials (PCM), sustainable materials for buildings, thermal energy storage

## Abstract

Eco-sustainable, low toxic and low flammable poly-ethylene glycol (PEG) was forced into flakes of the porous Lecce stone (LS), collected as stone cutting wastes, employing a very simple cheap method, to produce a “form-stable” phase change material (PCM). The experimental PCM was included in mortars based on different binders (hydraulic lime, gypsum and cement) in two compositions. The main thermal and mechanical characteristics of the produced mortars were evaluated in order to assess the effects due to the incorporation of the PEG-based PCM. The mortars containing the PEG-based PCM were found to be suitable as thermal energy storage systems, still displaying the characteristics melting and crystallization peaks of PEG polymer, even if the related enthalpies measured on the mortars were appreciably reduced respect to pure PEG. The general reduction in mechanical properties (in flexural and compressive mode) measured on all the mortars, brought about by the presence of PEG-based PCM, was overcome by producing mortars possessing a greater amount of binder. The proposed LS/PEG composite can be considered, therefore, as a promising PCM system for the different mortars analyzed, provided that an optimal composition is identified for each binder.

## 1. Introduction

At the present time, the worldwide research is strongly oriented to identify innovative routes to reduce the global consumption of energy. The concerns about the climate change, from one side, and the rapid depletion of natural resources, from the other, are pushing the international policy to invest on research activities that can address the growing pressing environmental issues, for instance, by improving energy efficiency of buildings. Buildings, in fact, are one of the biggest energy consumers, due to the internal heating/cooling requirements. In addition, the principal source of energy used in buildings comes from non-renewable fossil fuels, whose combustion develops carbon dioxide, with a consequent strong negative impact on the environment [1].

In ancient or old residential buildings low levels of thermal efficiency are frequently registered, since they have been constructed in absence of any regulation or rule from this point of view, neither employing proper insulation materials or devices [2]. Such buildings, therefore, need to be renewed in order to improve the thermal comfort conditions for occupants and to reduce the consumption of energy for heating and cooling; this task can be realized using innovative smart materials [3]. Among the recent techniques devised to improve energy efficiency in buildings, a relevant position is covered by the latent heat thermal energy storage (LHTES) systems, involving the storage of energy in a so-called phase change material (PCM) incorporated in construction materials.

A PCM is able to change its physical status, i.e., from liquid to solid to liquid again, as a consequence of the fluctuations in the external temperature [4]. When the environmental temperature is high (during daytime, for instance), a PCM is able to melt and store the melting enthalpy. In contrast, when the external temperature decreases, the PCM is capable to release the previously stored energy, solidifying again [5]. Due to this novel technology, the temperature inside a building can be maintained fairly constant, with a consequent decrease in heating/cooling energy expenses. The use of a PCM system in construction materials is able to supply several additional advantages, in terms of: reduced gaps between peak and off-peak thermal loads; cut in energy costs; improved interior thermal comfort in buildings and reductions of CO_2_ developed in atmosphere [6,7].

The incorporation of a suitable PCM into construction materials, through the passive building concept, has been recognized as the most effective solution. Different elements in a building (i.e., wallboard, floors, bricks, roof and concrete) can be combined with PCMs to increase their thermal energy storage capacity [8,9,10]. Other solutions can be also used in constructions with the same aim [11,12].

However, according to recent literature, the most feasible solution to include a PCM is based on its introduction inside the building envelope. In this way, the phase change material will be able to absorb and release heat during the hours of daylight [6,13,14,15,16,17].

Mortars, based on different binders, are considered as suitable mediums for PCMs. The incorporation of a PCM in a mortar represents a valuable solution due to large heat exchange area surfaces where mortars are applied. In addition, the PCM material included in the mortar can be shaped in a wide variety of forms and sizes, for each specific need [18]. The first experimental researches on mortars containing PCMs were largely focused on cement and gypsum binders, due to their initial good mechanical performance and thermal properties. However, when PCMs are added to such binders, substantial reductions in mechanical properties are generally registered [19,20,21].

Starting from these unsatisfactory results, further research moved towards investigation focused on mortars to be used as renders and coatings: these materials, in fact, do not require elevated values of mechanical strength. These mortars can be realized using different binders, such as aerial or hydraulic lime and, in some case, geopolymers [2,22,23]. Lime-based mortars, in addition, can be employed for building retrofitting, where render compatibility must be assured [24]. 

The form-stable is one of the easiest methods to incorporate an active PCM component into a porous inert support material [7,19]. The PCM composite can be, in fact, obtained by immersing the inert support in the liquid PCM; a vacuum pump can be employed to force the impregnation process.

In the first part of the research [25,26], a novel eco-sustainable form-stable polymeric PCM has been prepared, starting from small pieces of Lecce stone (LS), as support matrix, and low toxic and low flammable PEG (poly-ethylene glycol) as active phase change material. LS was obtained by waste product from a quarry sited in the same region where University of Salento is located. PEG possesses suitable phase change temperatures [26]; its large melting/crystallization enthalpy further supports the selection of this material. PEG displays also high long-term thermal/chemical stability and resistance to corrosion, with a limited volume change during solid–liquid phase transformation [27]. The originality of the designed PCM resides in the use of a waste natural material (LS) with the addition of an eco-sustainable one (PEG); the simplicity of the procedure used to obtain the PCM composite and the low cost of the resulting composite system represent additional valuable benefits.

In a first paper [26], the obtained LS/PEG form-stable PCM system was added as aggregate to an aerial lime, measuring different physical and mechanical properties of the resulting mortars. In this second paper, the same PCM composite was included to hydraulic lime, gypsum and cement-based mortar formulations. Taking into account the aim of the wide research project, i.e., the assessment of the thermal efficiency of the proposed novel PCM to manufacture mortars based on different binders, the influence of the PCM inclusion on some properties of the fresh and hardened mortars, such as workability, compressive and flexural strengths, was investigated.

## 2. Materials and Methods

### 2.1. Materials: LS/PEG Composite

**Figure 1:**
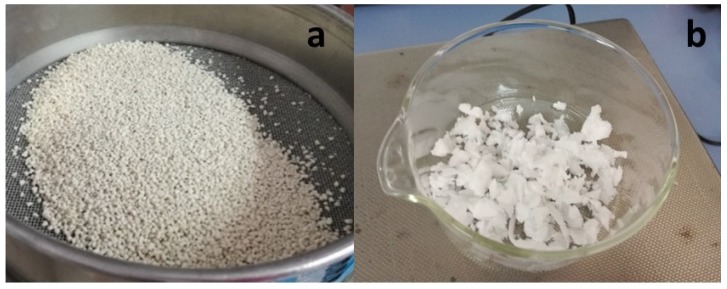
(**a**) Milled and sieved Lecce stone and (**b**) PEG 1000 in solid form.

Starting from our previous work [25,26], Lecce stone (LS), a biocalcarenite typical of Salento area (South Italy), was chosen as a porous support to realize form—stable PCM composites, to be added to different mortars. LS was specially selected for its characteristic high open porosity [28]; in addition, LS can be readily available as a waste product of the extraction and working of the stone from quarries. In this study, Lecce stone, supplied in the form of flakes, was further reduced in small pieces and sieved up to a granulometry ranging between 1.6 and 2.0 mm, as illustrated in Figure 1a. 

The PCM selected in this study was poly(ethylene glycol). It was supplied in solid form (Sigma–Aldrich company, Germany) with the trade name PEG 1000. According to the data sheet, the density of PEG 1000 at 20 °C was 1.2 g/cm^3^. The purchased product is illustrated in Figure 1b. The motivation with which PEG 1000 was selected in this research mainly relied on its favorable melting characteristics (a melting point ranging between 37 °C and 40 °C, and a heat of melting of about 129 J/g), which render this material a potentially optimal phase change material for mortars to be employed in Mediterranean regions [26,29,30]. PEG, in addition, displays a series of positive properties, such as: its cheapness, the low environmental impact and toxicity, and a low flammability, all features highly appreciated in the construction industry.

**Figure 2:**
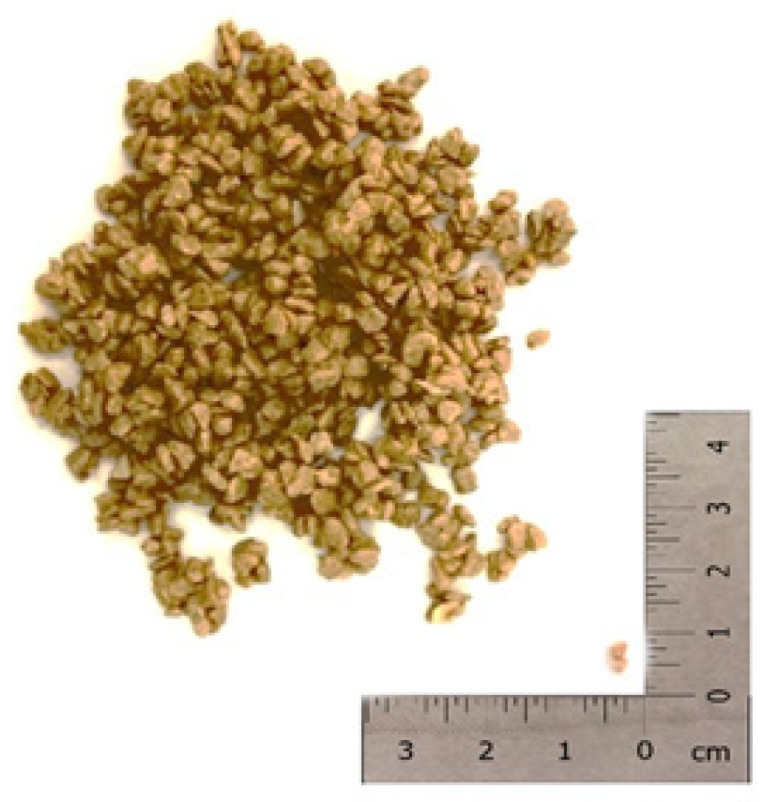
Form–stable LS/PEG composite obtained through the vacuum impregnation process.

To prepare the form—stable LS/PEG composite (as shown in Figure 2)—a vacuum impregnation process was employed, a cheap and simple method that can be easily realized in a small scale laboratory as well as at the industrial level. The detailed procedure employed, identified as the best one after several trials, has been reported in our previous work [26]; the percentage of PEG that is absorbed in LS, in these specific conditions, is 23% by weight. In the same paper, it was demonstrated that the sustainable LS/PEG stable-form PCM, produced following the optimized procedure, displayed appropriate LHTES properties and, therefore, it is a promising candidate to produce mortars able to improve the thermal efficiency of the buildings, increasing the comfort conditions of occupants.

### 2.2. Materials: Mortars and Their Manufacture

Different binders were employed in this study to produce mortars containing form-stable LS/PEG, i.e., hydraulic lime, gypsum and cement. In a previous paper, aerial lime-based mortar formulations containing this PCM have been already produced and investigated [26]. It was found, however, that the addition of the PEG-based PCM caused an unsuitable reduction of compressive and flexural strength values of the aerial lime-based mortar. The ongoing research project, therefore, continues the investigations in order to identify a different mortar, with an appropriate composition, that can take advantage from the addition of LS/PEG in terms of energy saving, still displaying adequate mechanical properties for the intended applications.

Different Portuguese companies supplied the following binders: a natural hydraulic lime (NHL) with a density of 2700 kg/m^3^, was supplied by CIMPOR (Lisbon, Portugal); a conventional gypsum, with high fineness and density of 2960 kg/m^3^, was provided by SIVAL (Souto da Carpalhosa, Leira, Portugal); finally, a CEM I 42.5 R cement, with a density of 3030 kg/m^3^, was supplied by SECIL (Lisbon, Portugal). The chemical composition of the cement was SiO_2_, Al_2_O_3_, Fe_2_O_3_, CaO, MgO, SO_3_, K_2_O and Na_2_O, with a specific surface area of 4007 cm^2^/g.

A superplasticizer (SP), i.e., a polyacrylate (MasterGlenium SKY 627, by BASF company), was always added to each mortar composition in the same quantity, in order to reduce the amount of water required for the mixing. The density of the SP is 1050 kg/m^3^. In Table 1, the composition of all the mortars realized and analyzed, produced according to the European Norm EN 998-1 [31], are reported. 

A total of twelve compositions were developed: six of them were produced by adding different percentages of LS/PEG to the binders, in order to evaluate the thermal properties of the single mortars as a function of the binder and of the PCM contents. For comparison purposes, six control formulations were prepared by introducing LS alone as aggregate. The indication “water saturation” in Table 1 accounts for the water used to saturate the LS aggregates, possessing a high porosity, to prevent them from absorbing the required water for the mortars manufacture. This additional water was not required when LS/PEG composite was added, since PEG was able to (almost) completely saturate the pores of Lecce stone.

Referring to the compositions selected to manufacture the mortars, it is well known that a certain reduction in mechanical properties of the mortars containing the PCM composite can be expected [32]. Starting from this consideration, some mortar compositions possessing a high amount of binder were also produced. The aim of the present study was, in fact, to identify the most convenient composition for each binder able to produce mortars with a good thermal efficiency and, at the same time, good mechanical properties.

### 2.3. Methods and Test Procedures

The workability in the fresh state of the produced mortars (summarized in Table 1) was first assessed. To this aim, the flow table method was employed, according to the European code EN 1015-3 [33]. The test was repeated twice, at least, on each produced formulation and the results averaged.

Then, the mechanical properties of the 28-days cured mortars (cast in iron molds, de-molded after 2 days and left for 26 additional days in standard conditions of 25 °C, R.H. of 50%), were measured in both flexural and compressive mode, following the standard EN 1015-11 recommendations [34]. For each composition of the different mortars systems, three prismatic specimens (40 × 40 × 160 mm^3^) were tested using a Lloyd dynamometer machine (LR50K Plus by Ametek Company), with a load cell of 50 kN, and the results averaged. The speeds employed to perform mechanical tests were 6 μm/s for flexural tests and 12 μm/s for compressive ones, respectively.

Calorimetry was employed to measure the phase change processes taking place in each mortar formulation containing the PEG-based PCM [25]. With this purpose, a DSC1 (Stare System, Mettler Toledo) instrument was employed to analyze small samples of each mortar if subjected to heating–cooling thermal cycles, performed at 10 °C/min under nitrogen atmosphere (flow rate: 60 mL min^−1^): the first from –10° to 100 °C, the second from 100° to –10 °C. For each mortar formulation, three specimens were analyzed, averaging the results. For comparison purposes, samples of the pure PEG were analyzed in DSC, using the same procedure previously described.

## 3. Results and Discussion 

### 3.1. Workability of Mortars

Firstly, the workability of the produced mortars was assessed; the values measured on each formulation are reported in Table 2. Keeping in mind that an appropriate value of workability for all the mortars produced (based on hydraulic lime, gypsum and cement) should lie in the range 160–180 mm [32], from the observation of data in Table 2 it is concluded that all the studied mortar formulations display an adequate value of workability.

**Figure 3:**
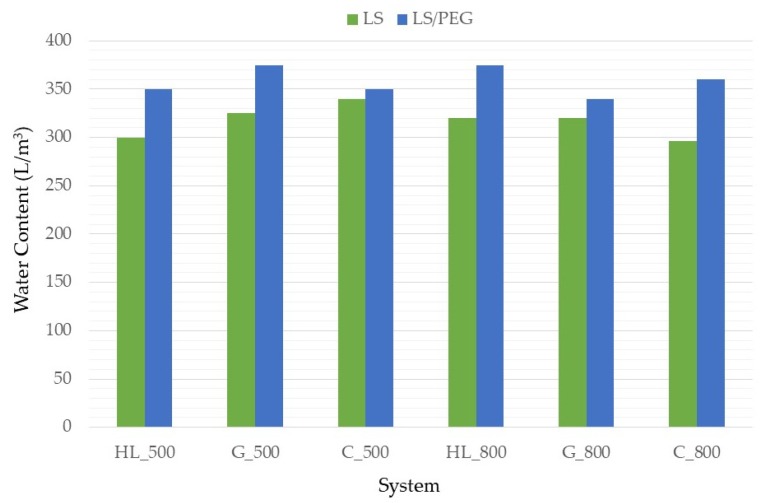
Variation of the water content for each mortar composition with the addition of the phase change material (PCM).

Sometimes, in order to reach a good workability, it is necessary to increase the amount of water of the mortar composition and, according to Figure 3, it was observed that the amount of water increase when the LS/PEG aggregates were used. 

The incorporation of PCM led to a rise in water content of about 15% for the hydraulic lime based mortar and for the gypsum; while for the cement based mortar was less, especially when it was used a higher content of binder. On the other hand, as reported in the literature, the incorporation of PCM causes an increase in the amount of water [21,35]. It is well known that a proper content of water is necessary to assure a good workability of the mortar formulation and that this amount should not be excessive: it can lead, in fact, to a high microporosity in the mortar and, as a consequence, to unsuitable mechanical properties [36]. Therefore, superplasticizers (SP) are commonly used as they improve the workability of the mortars even using limited amounts of water.

### 3.2. Results of DSC Analysis Performed on Mortars

**Figure 4:**
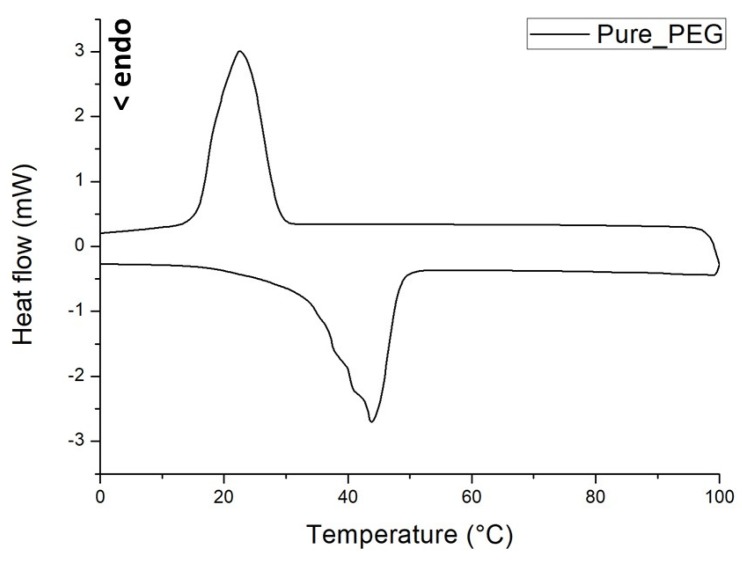
DSC thermograms recorded of pure PEG.

The DSC trace obtained from calorimetric analysis performed on pure PEG is shown in Figure 4.

As previously reported [26], the used PEG polymer exhibits an endothermic (melting) peak approximately at 43 °C during the heating stage and an exothermic (crystallization) peak around 23 °C when the temperature is reduced down. Melting/crystallization enthalpy of about 129 J/g was calculated from DSC measurements, in accordance with results of different studies performed on the same material [27,37]. As already underlined, the thermal characteristics displayed by PEG 1000, i.e., the phase change temperatures and enthalpies, are appropriate for developing a form-stable PCM to be used as thermal energy storage material. In fact, also the prepared LS/PEG composite exhibited thermal properties good enough to include this composite as an effective PCM in indoor mortars, especially for applications in buildings located in warm (for instance Mediterranean) regions [26].

**Figure 5:**
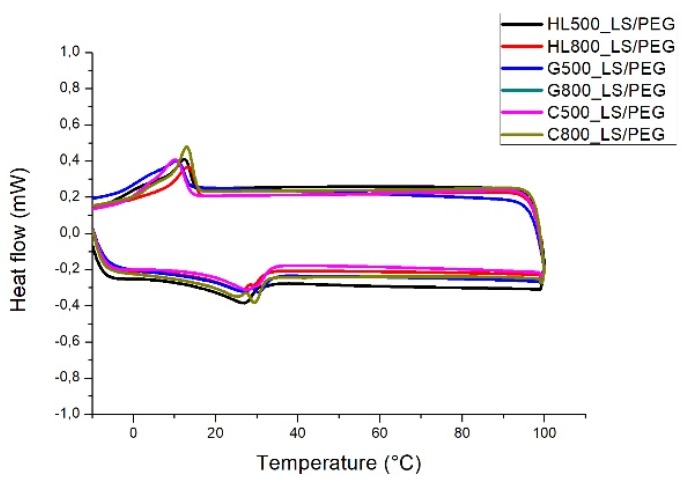
DSC thermograms of mortars containing LS/PEG PCM.

Then, in order to assess if LS/PEG aggregates can display suitable phase change characteristics even when incorporated into different mortars, DSC analyses were performed on small pieces of cured mortars containing the LS/PEG composite. The DSC traces recorded for mortar specimens containing LS/PEG composite are shown in Figure 5. The results obtained from the DSC experiments are summarized in Table 3.

The theoretical enthalpy (ΔH_Theor_) was also determined using Equation (1) [38].

ΔH_Theor_ = (PEG% × ΔH_PEG_)/100 (1)

where: PEG% is the PEG content in percentage and ΔH_PEG_ denotes the latent heat of the pristine PEG.

On the DSC thermograms of the mortars based on different binders and containing PEG added to Lecce stone (Figure 5), endothermic and exothermic peaks appeared during heating and cooling cycles, respectively, representing the melting and crystallization processes taking place in the PEG component. This observation confirms that phase transitions occurred in the mortar formulations containing the PCM under analysis, even if a low amount of the “active” component of the PCM is present [39]. Generally speaking, the higher the PCM content, the better the heat storage capacity of the mortar [40,41]. As expected, the specimens of mortars containing only LS did not display any melting/crystallization phenomena in the investigated range of temperatures (i.e., up to 100 °C).

The melting and crystallization peak temperatures calculated for the mortar specimens containing LS/PEG aggregates are in the range 27–30 °C and 10–13 °C, respectively, irrespective of the kind of binder. These temperatures are reported to be favorable to obtain a PCM-based mortar to be employed as thermal energy storage system included in the exterior and/or in indoor walls of buildings located in warm regions [35].

From the observation of the data reported in Table 3, it is noticed that both the melting and crystallization processes taking place in the PEG component contained in the mortars occurred at lower temperatures then those of pure PEG, being the decrease in the temperature peak approximately of 13–17 °C for melting process and of 10–13°C for the crystallization one. The shift in melting and crystallization processes toward lower temperatures has already found in different similar studies [25,38,42,43,44,45,46,47,48]. It has been attributed to physical surface interactions (such as capillary forces, hydrogen bonds and surface adsorption) between PEG and the other different components of each mortar.

Referring to the melting/crystallization enthalpies, the observed results, denoting a drastic decrease of the values (around 7–9 J/g) measured for the mortars containing PCM in comparison to the enthalpies of pure PEG (129 J/g), are mainly due to the low amount of PEG (that is the only crystallizable phase) present into the mortars samples. However, peak temperatures and enthalpy values lower than those expected (i.e., the theoretical values, normalized to the PEG content), were always measured. The presumed reason for these results may be that most of the PEG chains embedded in the stone pores could experience the phase changes only to a limited extent [49]. Phase transition temperatures and enthalpies decline, until the peak disappearance, as a consequence of confinement [50,51,52]; this occurs since the change from the crystalline to the melting state (and vice versa) is hindered. In the case of the investigated mortars, it can be hypothesized that, not only the store pore confinement influenced the thermal properties, but also the binder surrounding the LS/PEG aggregates can limit the PEG movements, thus further reducing the phase transition temperatures and enthalpies [53,54]. 

Nevertheless, the presence of well-defined and measurable melting/crystallization peaks also in the mortars containing the proposed PCM testifies that LS/PEG composite has a potential to act as an efficient phase change material.

### 3.3. Mechanical Properties of Mortars

The results of flexural and compressive mechanical tests performed on the different mortar formulations under study are reported in Table 4. 

As observed from the data reported in Table 4, an appreciable decrease in both flexural and compressive strength values is related to the introduction of LS/PEG composite and, most likely, to the greater content of water necessary when the PCM is added to the mortar compositions, irrespective to the kind of binder. It is reported, in fact, that the increase of water content in mortars upon addition of PCMs determines an increase in their microporosity, leading the latter to reductions in their mechanical strength [55]. In fact, the efforts of researchers moved towards the study of mortars basically employed for interior and/or exterior coatings, since a very high value of mechanical strength is not mandatory in such applications. It could be also hypothesized, however, that the decrease of the flexural and compressive strength values can be, at least partly, attributed to a loss of adhesion between the PCM aggregate and the binder paste. Analytical studies are in progress to prove, or exclude, this hypothesis. Finally, as expected, by increasing the percentage of binder, it is possible to increase the mechanical characteristics (flexural and compressive mode) even in the mortars containing LS/PEG composite [56].

The reductions in flexural strength brought about by the addition of PEG go from 62%, registered for G_800__LS/PEG system, to 95%, in the case of HL_500__LS/PEG. Referring to the compressive strength values, hydraulic lime and gypsum with a binder content of 500 kg/m^3^ present a decrease of one strength class when PEG is added to Lecce stone aggregate for the mortars, i.e., from CSII to CSI.

The reduction in compressive strength was much more appreciable in the case of cement-based mortar containing a binder content of 500 kg/m^3^ (about 94%), with a drop in the strength class classification from CSIV to CSI. By increasing the binder content up to 800 kg/m^3^, hydraulic lime presented a noticeable decrease in compressive strength by the incorporation of PEG, with a consequent strong reduction in the strength class classification, from CSIV to CSI/CSII. Referring to gypsum, a high decrease in compressive strength was again recorded (nearly 80%), with a consequent fall from CSIV to CSII classification. Similar results were found for cement-based mortars, with a higher decrease in compressive strength (about 87%) and a drop from CSIV to CSII strength class classification.

Keeping in mind that, to be successfully employed in the construction field, the mortars should respect the recommendations reported in the standard NP EN 998-1, thus they should have a minimum classification of CS II, the systems G_800__LS/PEG and C_800__LS/PEG are both fully respecting this requisite, while the formulation HL_800__LS/PEG is close to the strength target value. The mortars containing the PCM based on LS/PEG with a content of binder 500 kg/m^3^, on the other hand, display compressive characteristics not fully adequate (i.e., falling within CS I type).

Hydraulic lime is generally used to produce plastic mortars easily to apply and capable to set and harden in extreme conditions, including underwater, making this material appropriate for applications located close to the sea, lakes, rivers, etc. They are typically used in applications characterized by not excessive loads and to realize foundation mortars as well as renders and plasters for conservation, restoration and new build construction. An advantage is represented by its permeability to water vapor, i.e., it does not trap moisture in the walls allowing buildings to “breathe”.

Despite the fact that most of the research on mortars containing PCMs is focused on gypsum and cement formulations, due to the outstanding thermal characteristics and mechanical properties of these binders, some papers dealing with the use of phase change materials in hydraulic lime appeared in the last years [22,55,56,57,58]. Referring to the mechanical characteristics displayed by these modified-mortars, the addition of 20% and 40% microencapsulated PCM to hydraulic lime (500 kg/m^3^ content) determined a decrease in compressive strength of about 47% and 52% (the latter from 5.37 MPa for the hydraulic lime control system to 2.58 MPa), respectively [55,56]. While, the decrease in flexural strength of the hydraulic lime mortar including 20% or 40% of PCM microcapsules was found around 20% and 30%, respectively. Similar results were found in a different study again focused on hydraulic lime mortar, again with the addition of a 40% microencapsulated PCM, by the same authors, with a decrease in flexural strength of about 27% (from 2.2 MPa for control mortar to 1.6 MPa for composite formulation) [58].

In conclusion, the results of mechanical characteristics (flexural and compressive strength) found in the present study for hydraulic lime mortar containing LS/PEG composite, when compared with the same mortar modified with different PCM systems, appeared to be fairly satisfactory for the proposed applications.

Gypsum is one of the more widely used construction materials, mainly in interior designing. It is mainly used as surface materials, being its application prominent for finishing wall and ceiling in form of plaster. Gypsum is light, long-lasting and presents an intrinsic very high fire resistance.

There are several papers published in the last years on gypsum-based mortars containing different PCM systems [55,56,58,59,60,61]. Even for this kind of binder, the good mechanical properties of gypsum were found to be severely compromised by the addition of a PCM system. In fact, it was found that the introduction of 20% and 40% of PCM microcapsules determined a decrease in the flexural strength of gypsum (500 kg/m^3^ content) of around 40% and up to 80%, respectively, and in compressive strength of about 50% and 64%, respectively [55,56,58]. The reductions in strength found in our research for PCM-modified gypsum, therefore, are more or less in line with those reported in previous literature, especially if we refer to the gypsum mortar containing the highest content of binder, i.e., 800 kg/m^3^. It is confirmed, therefore, that we can consider this system suitable for the proposed applications.

The use of cement-based mortars in constructions is extremely wide: from restoration and repairing of damaged concrete to the patching or filling concrete, rendering and floor leveling; it can also be used to produce precast products. These mortars display a great mechanical resistance and a low porosity, with consequent impermeability to water vapor.

The reductions in flexural strength values calculated on mortars based on cement (CEM I type) and containing a PCM system achieved up to 50% depending on the kind and amount of PCM system [56]. The reductions in compressive strength are even more severe (up to 70%) at the highest contents of encapsulated PCM [20,21,56,62]. Our cement-based mortars containing LS/PEG PCM, therefore, followed the same trend, once again referring to the mortar containing the highest content of cement, i.e., 800 kg/m^3^.

From the results of mechanical (flexural and compressive) tests, and the relative discussion, it can be concluded that all the produced mortar compositions display values of flexural and compressive strengths that are included in the standard classification for not structural mortars. On the other hand, not all of them fall into the CSII type based on the compressive strength, as requested by the standard NP EN 998-1. Adequate mechanical properties for the intended purpose are those recorded for the gypsum- and cement-based mortars containing a greater amount of binder (800 kg/m^3^); close to the strength target value is also the formulation HL_800__LS/PEG. None of the mortars containing the PCM based on LS/PEG with a content of binder 500 kg/m^3^ falls within the CS I type.

Experiments are in progress on some of the produced formulations using the largest amount of binder (800 kg/m^3^), in order to verify the thermal properties of such formulations and, in turn, to assess the effective efficiency as phase change material of LS/PEG composite. 

## 4. Conclusions

A wide experimental investigation was carried out in order to evaluate the effect of the incorporation of a PEG-based PCM on the mechanical and thermal properties of mortars based on different binders. The selected PCM was composed by eco-sustainable PEG simply included in a waste of natural stone, i.e., Lecce stone. The low cost of the method used to produce the PCM composite, low toxicity and low flammability of both components constitute advantageous additional characteristics for applications in the construction industry.

The obtained results showed that the addition of a phase change material in mortars employed for the thermal isolation of building walls (for indoor/outdoor applications) caused significant changes in their properties, either in the fresh and hardened state. As expected, the incorporation of LS/PEG aggregates required an increase in the amount of water to reach a suitable workability in all mortars. It was verified that the thermal properties of the LS/PEG composite decreased when it was incorporated as an aggregate in mortar mixes. However, well-defined and measurable melting/crystallization peaks were still observed in the mortars containing the proposed PCM. The melting and crystallization peak temperatures allowed for obtaining PCM-based mortars suitable as thermal energy storage systems in both exterior and indoor walls of buildings, especially those located in warm regions. The mechanical properties (in flexural and compressive mode) decreased when LS/PEG aggregates were included in the mortar compositions. This phenomenon, however, is common for any mortar containing a PCM system, as reported in the literature. Nevertheless, by increasing the amount of binder, it was possible to achieve mortars that can be classified at least as CSII, except for the system based on hydraulic lime, i.e., HL800LS/PEG. Even this system, however, can be classified in between CSI and CSII categories. 

In order to assess if the produced PCM-mortars are effectively able to produce an improvement in the thermal performance of building walls realized with them, and consequently to limit the energy consumption for cooling and heating, experiments are in progress to evaluate the thermal performance of elements produced with such novel mortars.

## Figures and Tables

**Table 1:** Mortar compositions (reported as kg/m^3^ of produced mortar).

System	Binder/Content	Aggregates		SP	Water Saturation	Water	Water/Binder
		LS	PEG Content				
HL_500__LS	Hydraulic Lime/500	1480	0	15	370	300	0.60
HL_500__LS/PEG	Hydraulic Lime/500	1678	386	15	0	350	0.70
G_500__LS	Gypsum/500	1454	0	15	366	325	0.65
G_500__LS/PEG	Gypsum/500	1645	378	15	0	375	0.75
C_500__LS	Cement/500	1392	0	15	350	340	0.68
C_500__LS/PEG	Cement/500	1790	412	15	0	350	0.70
HL_800__LS	Hydraulic Lime/800	1092	0	15	275	320	0.40
HL_800__LS/PEG	Hydraulic Lime/800	1729	398	15	0	375	0.47
G_800__LS	Gypsum/800	1169	0	15	294	320	0.40
G_800__LS/PEG	Gypsum/800	1472	339	15	0	340	0.43
C_800__LS	Cement/800	1070	0	15	269	296	0.37
C_800__LS/PEG	Cement/800	1347	310	15	0	360	0.45

**Table 2:** Values of workability of the mortars possessing different compositions.

System	Workability (mm)
HL_500__LS	170 ± 2
HL_500__LS/PEG	177 ± 3
G_500__LS	160 ± 1
G_500__LS/PEG	180 ± 3
C_500__LS	160 ± 1
C_500__LS/PEG	160 ± 1
HL_800__LS	165 ± 2
HL_800__LS/PEG	175 ± 2
G_800__LS	160 ± 1
G_800__LS/PEG	160 ± 1
C_800__LS	160 ± 1
C_800__LS/PEG	178 ± 3

**Table 3:** Characteristic (initial, end and peak) temperatures and enthalpy measured during heating stage (melting) and subsequent cooling stage (crystallization) on pure PEG, on LS/PEG composite and on mortars, based on different binders, containing LS/PEG composite. For each investigated system, the content in PEG as a percentage and the theoretical enthalpies (as defined in Equation (1)), for both melting (ΔHm _Theor_) and crystallization (ΔHc _Theor_), are reported.

	**System**	**PEG Content (%)**	**Onset (°C)**	**Endset (°C)**	**Tm (°C)**	**ΔHm (J/g)**	**ΔHm _Theor_ (J/g)**
**Heating Stage**	PEG	100	36.4 ± 0.6	50.2 ± 0.3	42.8 ± 1.1	129.3 ± 1.2	---
	LS/PEG	23	12.4 ± 0.5	50.2 ± 0.8	39.3 ± 0.7	27.7 ± 0.9	29.7
	HL_500__LS/PEG	15.2	9.7 ± 0.4	37.9 ± 0.6	26.9 ± 0.7	6.8 ± 1.1	19.7
	HL_800__LS/PEG	13.6	2.2 ± 1.0	41.8 ± 1.1	26.0 ± 0.8	7.9 ± 0.9	17.6
	G_500__LS/PEG	14.9	3.5 ± 0.4	42.8 ± 0.2	30.4 ± 0.4	8.5 ± 0.9	19.3
	G_800__LS/PEG	12.9	2.7 ± 0.5	35.9 ± 0.7	28.9 ± 1.0	7.8 ± 1.2	16.7
	C_500__LS/PEG	15.5	4.3 ± 1.1	38.4 ± 0.7	27.6 ± 0.3	9.0 ± 1.3	20.0
	C_800__LS/PEG	12.3	3.4 ± 0.1	36.3 ± 0.1	30.0 ± 0.3	7.7 ± 0.2	15.9
	**System**	**PEG Content (%)**	**Endset (°C)**	**Onset (°C)**	**Tc (°C)**	**ΔHc (J/g)**	**ΔHc _Theor_ (J/g)**
**Cooling Stage**	PEG	100	18.3 ± 1.1	26.7 ± 1.1	23.6 ± 1.2	129.8 ± 0.8	---
	LS/PEG	23	3.7 ± 0.1	28.8 ± 0.3	19.4 ± 0.2	28.6 ± 0.1	38.6
	HL_500__LS/PEG	15.2	0.4 ± 0.5	17.6 ± 0.3	12.5 ± 0.4	6.2 ± 1.1	25.5
	HL_800__LS/PEG	13.6	1.6 ± 0.4	18.9 ± 0.8	13.5 ± 0.2	6.0 ± 0.7	22.8
	G_500__LS/PEG	14.9	−1.1 ± 0.6	19.2 ± 0.7	10.6 ± 0.8	7.4 ± 0.9	25.0
	G_800__LS/PEG	12.9	0.6 ± 0.9	16.9 ± 1.0	10.8 ± 0.7	7.5 ± 1.2	21.7
	C_500__LS/PEG	15.5	0.2 ± 0.6	19.2 ± 0.7	10.3 ± 0.3	8.8 ± 1.2	26.0
	C_800__LS/PEG	12.3	−1.1 ± 0.2	16.7 ± 0.2	10.8 ± 0.3	8.7 ± 0.4	20.6

**Table 4:** Mechanical properties of the cured mortars measured in flexural and compressive mode.

System	Flexural Strength (MPa)	Compressive Strength (MPa)
HL_500__LS	1.1 ± 0.3	2.8 ± 0.8 [CSII]^a^
HL_500__LS/PEG	0.1 ± 0.1	0.4 ± 0.1 [CSI] ^a^
G_500__LS	3.2 ± 0.2	4.8 ± 0.2 [CSII] ^a^
G_500__LS/PEG	0.5 ± 0.1	0.4 ± 0.2 [CSI] ^a^
C_500__LS	5.8 ± 0.3	20.5 ± 0.4 [CSIV] ^a^
C_500__LS/PEG	1.0 ± 0.2	1.1 ± 0.2 [CSI] ^a^
HL_800__LS	2.8 ± 0.5	17.0 ± 0.2 [CSIV] ^a^
HL_800__LS/PEG	0.4 ± 0.1	1.5 ± 0.1 [CSI/CSII] ^a^
G_800__LS	4.1 ± 0.2	16.4 ± 0.6 [CSIV] ^a^
G_800__LS/PEG	1.6 ± 0.2	3.3 ± 0.3 [CSII] ^a^
C_800__LS	9.2 ± 0.9	26.3 ± 0.4 [CSIV] ^a^
C_800__LS/PEG	1.9 ± 0.3	3.4 ± 0.8 [CSII] ^a^

^a^ Category of the mechanical resistance of the mortar according to the standard NP EN 998-1.
